# Enzymatic Processing of DNA–Protein Crosslinks

**DOI:** 10.3390/genes15010085

**Published:** 2024-01-10

**Authors:** Maram M. Essawy, Colin Campbell

**Affiliations:** Department of Pharmacology, University of Minnesota, Minneapolis, MN 55455, USA; essaw002@umn.edu

**Keywords:** DNA–protein crosslink (DPC), direct crosslink reversal, nuclease, protease, ubiquitin, SUMO, poly(ADP) ribose (PAR), proteasome, SPRTN

## Abstract

DNA–protein crosslinks (DPCs) represent a unique and complex form of DNA damage formed by covalent attachment of proteins to DNA. DPCs are formed through a variety of mechanisms and can significantly impede essential cellular processes such as transcription and replication. For this reason, anti-cancer drugs that form DPCs have proven effective in cancer therapy. While cells rely on numerous different processes to remove DPCs, the molecular mechanisms responsible for orchestrating these processes remain obscure. Having this insight could potentially be harnessed therapeutically to improve clinical outcomes in the battle against cancer. In this review, we describe the ways cells enzymatically process DPCs. These processing events include direct reversal of the DPC via hydrolysis, nuclease digestion of the DNA backbone to delete the DPC and surrounding DNA, proteolytic processing of the crosslinked protein, as well as covalent modification of the DNA-crosslinked proteins with ubiquitin, SUMO, and Poly(ADP) Ribose (PAR).

## 1. Introduction

DNA–protein crosslinks (DPCS) result from the covalent trapping of proteins onto DNA [1,2]. Proteins become covalently bound to DNA through a variety of mechanisms, broadly categorized as enzymatic or non-enzymatic. These categories of DPC formation are described extensively in several different review articles [1,3,4,5]. Broadly, enzymatic mechanisms of DPC formation result from the trapping of transient DNA–protein interactions formed as part of the catalytic mechanism of DNA-interacting proteins [6,7,8,9]. Examples of these proteins include DNA polymerases and numerous proteins involved in DNA damage recognition and repair. Notably, polymerase β often becomes trapped in DNA during attempted removal of the oxidized abasic (AP) site 2-deoxyribonolactone (dL) [7,10]. Additionally, DNA topoisomerases involved in helix unwinding during DNA repair, notably Topoisomerase-1 (TOP-1) and Topoisomerase-2 (TOP-2) often become covalently trapped to the DNA backbone, as well as the DNA repair proteins Poly(ADP-Ribose) Polymerase 1 and 5-hydroxymetylcytosine (fhmC) binding, ESC specific (HMCES) [6,11,12,13,14,15,16,17,18]. Non-enzymatic DPCs form following cellular exposure to endogenous or exogenous reactive compounds that react with the DNA and/or nearby proteins, causing these proteins to become irreversibly bound to the DNA [19,20,21,22,23,24,25,26]. Commonly trapped proteins are histones, which are constantly in close proximity to DNA due to their involvement in the structural organization of the nuclear genome [27,28]; however, numerous other proteins are known to become crosslinked to chromosomal DNA [24,29]. Examples of endogenous reactive compounds include aldehydes and reactive oxygen species formed as byproducts of cellular processes. Examples of exogenous sources of DNA damage include UV radiation, ionizing radiation, and industrial chemicals like formaldehyde and 1,2,3,4 Diepoxy-butane [23,30,31,32,33,34,35]. While there are many chemical mechanisms by which these agents can form DPCs, certain DNA and protein residues tend to be more reactive, and thus act as focal points for DPC generation. For example, bis-electrophilic molecules can react with the N7 atom of guanine to generate a product that can subsequently combine with nucleophilic amino acid residues like cysteine of nearby proteins, resulting in the formation of a DNA protein crosslink. UV irradiation can form purine and pyrimidine free radicals, which then form covalent bonds with nearby proteins [36,37]. Similarly, ionizing radiation induces unstable DNA radical cations as well as protein radicals, that can react and form a covalent bond. Aldehydes like formaldehyde react with primary amines of lysine residues, which then react with the amino groups of aromatic DNA bases, and during this reaction, the transient Schiff base can transform into a covalent interaction, resulting in a DPC [38]. Reactive oxygen species (ROS) oxidize DNA nucleobases, which also can form a Schiff base with lysine residues of DNA-interacting enzymes that can be converted into a stable covalent bond [39,40]. Another source of exogenous DNA damage includes chemical compounds used in cancer chemotherapy, in fact, DNA-damaging drugs have proven highly effective in the treatment of several cancers, including breast, ovarian, and colorectal cancers [41,42]. While DNA-damaging drugs can form several different kinds of DNA damage, some of these chemotherapeutic agents form DPCs as well (these drugs will be described in more detail later in this passage [24,25,27,43,44,45,46,47,48].

DPCs are large and bulky, and for this reason, cause steric hindrance of essential cellular processes such as transcription and replication, and, if unrepaired, DPCs formed by these drugs ultimately result in cellular toxicity [49,50,51,52,53,54]. As any protein in the vicinity of DNA can theoretically be trapped onto DNA following exposure to these drugs, the size, structure, and chemical identity of DPCs formed by DPC-forming drugs can vary widely, as has been discussed in several DPC review articles [5,55,56]. Our group has used mass spectrometry-based analysis of DPCs formed in human cells to identify over 300 proteins that become crosslinked to chromosomal DNA following cisplatin or nitrogen mustard treatment [24,53]. Others have identified a variety of proteins crosslinked to DNA following treatment with ionizing radiation, including actin and histone H2B [22]. Notably, due to their mechanism of action, some DPC-forming drugs are only able to trap one type of protein onto DNA. For example, the topoisomerase inhibitors etoposide and camptothecin act as topoisomerase poisons that stabilize the transient bond formed between topoisomerase and DNA following the formation of a strand break in DNA by topoisomerase, and, for that reason, only topoisomerases are subject to trapping onto DNA following treatment with these drugs [45,57,58,59]. Following uptake into cells, the anti-cancer drug decitabine, 5-aza-2′-deoxycytidine (aza-dC), indicated in the treatment of myelodysplastic syndrome, undergoes phosphorylation and becomes incorporated into chromosomal DNA. When cellular DNA-methyltransferase (DNMT) recognizes this incorporated 5-aza-deoxycytidine residue and initiates a methylation reaction, the ordinarily transient reaction intermediate formed between the enzyme and deoxycytidine base cannot be resolved and, consequently, a DPC is formed between the enzyme and the chromosomal DNA. Due to this specific reaction mechanism, only DNA methyltransferases (DNMT1 primarily, and to a substantially lesser extent, DNMT3A and DNMT3B) are the only proteins that become crosslinked to DNA following exposure to aza-dC [47,60,61].

**Figure 1:**
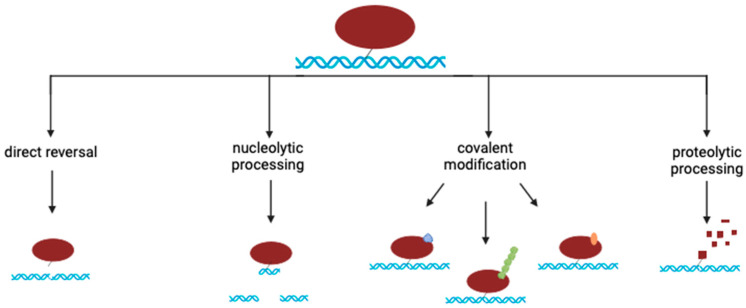
Enzymatic processing of DNA–protein crosslinks. The four types of enzymatic processes that modify DPCs are depicted. Direct reversal involves the hydrolysis of the covalent bond between the DNA and the crosslinked protein (catalyzed by proteins like TDP1 and TDP2). Nucleolytic processing involves the direct incision or excision of the DNA surrounding the DNA protein crosslink (catalyzed by nucleases like Mre11 and CtIP). The covalent modification involves the covalent attachment of proteins like SUMO (blue), ubiquitin (green), or chemical groups like ADP-Ribose (orange) as monomers or polymers onto the crosslinked protein (catalyzed by proteins like Ubiquitin E3 Ligase, SUMO E3 Ligase, and PARP1). Proteolytic processing involves the proteolytic digestion of the DNA-crosslinked protein (catalyzed by the proteasome or proteases like SPRTN and ACRC).

Presumably due to the inherent diversity in size, structure, and chemical bonds that crosslink proteins to chromosomal DNA, cells possess several distinct mechanisms through which they recognize and remove/repair DPCs. Broadly, some of these mechanisms result in the metabolism of the covalent crosslink between protein and DNA, some nucleolytically process the DNA surrounding the crosslink, and some proteolytically process tor covalently modify the DNA-crosslinked protein (as depicted in Figure 1). In this review, we briefly summarize a number of recent studies that have described the enzymatic machinery involved in these distinct types of DPC processing.

## 2. Enzymatic Processing of DPCs

### 2.1. Direct Crosslink Removal

As discussed above, proteins that regularly form transient bonds with DNA as part of their enzymatic interactions may become covalently trapped in the DNA and thus cells have evolved specialized repair mechanisms for the removal of these DPCs. These specialized repair mechanisms are mobilized to the direct removal of the chemical crosslink between the DNA and the DNA-interacting protein. The most studied example of this type of DPC processing is that of the removal of DNA-crosslinked topoisomerases, which can become trapped in the DNA backbone during failed DNA replication or following cellular treatment with chemicals that stabilize the DNA–topoisomerase complex. Tyrosyl-DNA phosphodiesterase 1 (TDP1) is a highly conserved repair enzyme dedicated to the excision of TOP-1 DPCs [62]. As TOP-1 DPCs form through a phosphotyrosyl linkage between the DNA backbone and the catalytic tyrosyl residue of TOP-1, TDP1 functions by directly targeting and hydrolyzing this phosphotyrosyl bond, resulting in the release of TOP-1 from the DNA backbone [6,58,63,64,65,66]. Similarly, TDP2 hydrolyzes the phosphotyrosyl bonds between TOP-2 and the DNA backbone [14,59,62,67]. Subsequent repair of the resulting single- or double-strand DNA breaks is discussed in Section 2.5. The 5-hydroxymetylcytosine (fhmC) binding, embryonic stem cell-specific (HMCES) protein can also become covalently attached to apurinic or apyrimidinic sites in single-stranded DNA. This crosslinking appears to prevent chromosomal DNA double strands from forming during replication. Interestingly, the crosslinked HMCES protein is able to catalyze a self-reversal reaction, resulting in the release and regeneration of free HMCES [15,68]. Interestingly, HMCES DPCs have also been implicated as intermediates in the repair of DNA–DNA interstrand crosslinks [16].

### 2.2. Nucleolytic Processing

Enzymatic modification of DPCs may alternatively involve nucleolytic cleavage of DNA flanking the DPC. This is exemplified in the case of spo11, an evolutionarily conserved protein that is related to archaebacterial topoisomerases [69,70,71] and is essential for the initiation of meiotic recombination in several species, including humans [72,73,74,75]. Like topoisomerase, spo11 forms a phosphotyrosyl linkage with the DNA backbone (however, unlike topoisomerase, this occurs following the dimerization of two spo11 proteins). The formation of a phosphotyrosyl linkage between the spo11 dimer and the DNA backbone results in the formation of a double-strand break in the backbone which is required for the initiation of meiotic homologous recombination. As spo11 is covalently bound to the 5′ phosphate of this DSB, spo11 must be removed prior to the initiation of homologous recombination. Release of chromosomal DNA-crosslinked spo11 occurs through endonucleolytic cleavage by mre11, resulting in the release of spo11 that remains covalently bound to a short oligonucleotide fragment, and the formation of protein-free chromosomal DNA with double-strand break ends at which meiotic recombination is initiated [73,75,76,77].

Nuclease-dependent processing of the DNA backbone resulting in the removal of a DPC has thus been shown to be required for the initiation of meiotic recombination; however, there is evidence to suggest that this type of enzymatic processing is involved in other types of (meiosis-independent) DPC repair as well. For example, Depshande et al. showed, using a DPC substrate in which streptavidin-bound biotin was linked to the 5′ end of a double-stranded DNA molecule, that Mre11-dependent nucleolytic processing of the DNA resulted in DPC removal in vitro [78]. This finding is supported by studies that have shown that yeast mutants deficient in Mre11 nuclease activity are sensitive to ionizing radiation [79]. C-terminal binding protein-interacting protein (CtIP), which is known to interact with the Mre11-Nbs1-Rad50 (MRN) complex, also has nuclease activity and was shown to promote the removal of topoisomerase 2 adducts in vitro (in a mechanism dependent on its nuclease activity) [80,81]. The resulting DNA double-strand breaks are believed to be subject to both recombinational and DNA end-joining repair pathways (see below). It is conceivable that other DNA nucleases may participate in DPC removal, however none have yet been identified.

### 2.3. Proteolytic Processing

#### 2.3.1. Proteasome

The first evidence that DPCs are subject to proteolytic degradation came from a study that showed that cellular treatment with lactacystin, an inhibitor of the proteasome, impaired the removal of formaldehyde-induced DPCs [35]. Since then, a number of additional studies have confirmed that the proteasome plays a role in the removal of drug-induced DPCs. Pharmacological inhibition of the proteasome using drugs like MG132 and bortezomib resulted in impaired removal of DPCs formed following cellular treatment with nitrogen mustards, topoisomerase inhibitors, or aza-dC [14,82,83,84,85]. Some studies have shown that following recognition of a DPC during replication, proteasomal degradation of DPCs resulted in the formation of DNA–peptide adducts that are then bypassed by trans-lesion synthesis mediated by an error-prone DNA polymerase [86]. Several groups have also shown that pharmacological inhibition of the proteasome impaired DPC repair [14,82,83,84,85,87,88,89,90,91,92,93]. However, other groups have failed to observe the effect of proteasome inhibition on DPC repair [94,95,96]. This apparent paradox may be explained in a number of ways. For example, it is conceivable that there is redundancy in the cellular proteases that are mobilized to remove DPCs. Consequently, these alternative processes can carry out DPC removal and thus have no net effect on DPC repair when the proteasome is inactivated. Consistent with this view, our group has observed cells can utilize either homologous recombination (HR) or nucleotide excision repair (NER) to repair the same DPC lesion. Interestingly, the two pathways appear to be functionally redundant, i.e., the efficiency of DPC repair was not diminished in cells in which either the HR or the NER pathways were inactive. Importantly, the inactivation of both pathways essentially eliminated DPC repair altogether [93,97]. Alternatively, it is possible that proteasomal degradation may be linked to the repair pathway mobilized to repair some DPC lesions, not others. The latter possibility is in line with studies that have shown that the NER machinery can directly initiate DNA incision around the DPC when DNA-crosslinked protein or peptide is smaller than 10–14 kDa [88]. In contrast, the Paull group showed that the Mre11-Rad50-Xrs2 protein complex was capable of inducing a DNA double-strand break adjacent to a streptavidin–DNA crosslink (molecular weight of streptavidin is ~60 kDa) [78]. Consistent with this latter interpretation, our lab has shown that a synthetic DPC substrate transfected into mammalian cells was subject to proteasome-dependent removal during NER-dependent DPC removal, but that HR-dependent DPC removal occurred via a proteasome-independent mechanism [93]. It is noteworthy that the protein component of this DPC (~42 kDa) was also considerably larger than the 10–14 kDa cut-off for NER-dependent DPC excision.

#### 2.3.2. Wss1 and SPRTN 

Wss1, a metalloprotease found in yeast was the first DPC-specific protease to be discovered [98,99]. Studies showed that wss1 is directly involved in the degradation of bothTop1cc complexes as well as formaldehyde-induced DPCs, and that clones lacking wss1 were hypersensitive to formaldehyde-induced cell death [98,100]. Soon after the identification of wss1 in yeast, the molecular mechanism was discovered for SPRTN, its mammalian homolog [101]. In a study of formaldehyde-induced DPCs, DNA-crosslinked protein removal was shown to be SPRTN dependent [95,102]. Most of the research implicates SPRTN in replication-coupled repair, which is supported by the finding that SPRTN is a constitutive component of the replisome [103,104,105]. SPRTN also plays a role in the orchestration of the response to stalled replication forks, including the modulation of translesion synthesis following cisplatin or UV-induced DNA damage [106,107,108,109,110]. Interestingly, however, it was shown in *Xenopus laevis* extracts that a DPC present on single-stranded DNA was subject to SPRTN-mediated removal even in the absence of a full replisome, suggesting that SPRTN can also be involved in replication-independent repair mechanisms [94,111]. Additionally, Kroning et al. showed that SPRTN-dependent DPC degradation in vitro occurred in the absence of replication-associated machinery or mechanisms, providing further evidence that SPRTN plays a role in replication-independent DPC degradation [112]. Together, these findings suggest that SPRTN is involved in replication-independent mechanisms through a process or processes that are not as well understood as replication-coupled SPRTN-dependent DPC removal. Various studies suggest that SPRTN and wss1-mediated degradation of DNA-crosslinked proteins promote polymerase bypass of the lesion during DNA replication, as will be discussed in Section 2.5 [94,98,113].

#### 2.3.3. Other Proteases

In a study of formaldehyde-treated *Caenorhabditis elegans*, the ACRC protease (also referred to as GCNA) was also implicated in the removal of formaldehyde-induced DPCs [102]. More recently, the protease Ddi1 in yeast was shown to contribute to the removal of stabilized TOP1 cleavage complexes in yeast. It should be noted, however, that it is not yet known whether the mammalian homologs DDI1 and/or DDI2 play a role in DPC removal [114]. Interestingly, the proteolytic activity of the human protease FAM111A was shown to protect cells from replication fork stalling at PARP1-DNA covalent complexes, suggesting that the FAM111A protease family is also involved in DPC proteolysis [115,116]. The discovery of additional proteases involved in DPC removal explains the findings by some research groups that proteasomal inhibition does not impair DPC repair, as the presence of several DPC processing proteases suggests that the proteasome is involved redundantly one or several of the proteases discussed above [86,117].

### 2.4. Covalent Modification

#### 2.4.1. Ubiquitination 

Ubiquitin is an 8.5 kDa protein that is evolutionarily conserved across nearly all eukaryotic organisms [118]. Ubiquitin can be conjugated to target proteins via one of its seven lysine residues in a process known as ubiquitination or ubiquitinylation [118,119,120]. While proteins can be ‘monoubiquitinated’, i.e., modified with one ubiquitin protein, they can also be polyubiquitinated, or modified with polyubiquitin chains formed following the linkage of multiple ubiquitin proteins to one another via the lysine residues of ubiquitin [121,122,123]. The most commonly formed and best-understood polyubiquitin chains are comprised of K48 and K63 polyubiquitin linkages; however, other polyubiquitin chains form via residues K6, K11, K27, K29, and K33 [122,123,124]. Distinct types of ubiquitination, whether monoubiquitination or different types of polyubiquitination, appear to trigger distinct cellular fates [125]. For example, monoubiquitination has been shown to result in the endocytic transport of various modified proteins [126,127,128]. K48 polyubiquitination is known to induce proteasomal degradation of the ubiquitinated protein, while K63 polyubiquitination is involved in multiple processes including DNA damage response signaling and immune signaling [124,129,130,131,132,133,134]. It is thus conceivable that polyubiquitination of DNA-crosslinked proteins can play a role in their removal and repair, and the findings that support this speculation are described below.

It was first shown that DPCs formed by anti-cancer drugs are post-translationally modified when a Western blot of TOP-1 DPCs recovered from camptothecin-treated C3H mouse mammary carcinoma cells revealed that the recovered TOP1 DPCs formed a distinct ladder of higher molecular weight bands resembling a ubiquitin ladder, and that this ladder was only observed with TOP1 that had been covalently crosslinked to DNA [87]. Later, treatment of various mammalian cell lines with camptothecin, a pharmacological inhibitor of topoisomerase 1 resulted in the formation of TOP1-ubiquitin conjugates [92,135,136]. DPCs formed following cellular treatment with *N*-methyl-2,2-di(chloroethyl)amine, aza-dC, and formaldehyde were all shown to be modified with ubiquitin [82,84,85,95,102]. Notably, studies have shown that post-translational modifications of DNA-crosslinked protein may drive further enzymatic processing of the DPC, including direct reversal and proteolytic degradation). For example, inhibition of the formation of K48 and K63 polyubiquitin chains impaired the removal of TOP1 DPCs, as well as the regulation of TDP2 catalytic activity [14,91,92]. Others showed that etoposide treatment increased TOP2α and TOP2β ubiquitination, and this effect was potentiated upon cotreatment with MG132, suggesting that the proteasome plays a role in the removal of covalently modified topoisomerase [14]. In formaldehyde-treated cells, replication-dependent localization of SPRTN to DPCs was impaired by pharmacological inhibition of ubiquitination [95,102]. Kroning et al. generated a model DPC substrate by fusing a di-ubiquitin moiety to a DPC substrate which was crosslinked to a DNA oligonucleotide containing a 5-base overhang that is specifically targeted by SPRTN. Using this DPC substrate, it was shown that tightly folded proteins that are crosslinked to DNA are first unfolded by the AAA+ type ATPase p97, which then facilitates the degradation of the crosslinked protein by SPRTN, which is unable to degrade tightly folded proteins [112]. Together, these studies suggest that ubiquitination plays a role in the removal of DNA-crosslinked proteins. While multiple types of polyubiquitination have been found to occur on DPCs, not all types have been thoroughly interrogated in this context (as described above, proteins may undergo mono-, multi-, or different types of polyubiquitination, and each of these types have multiple downstream roles); therefore, the exploration of the role of ubiquitination in DPC repair remains a compelling and dynamic area of inquiry.

#### 2.4.2. SUMOylation

Small Ubiquitin-like Modifier (SUMO) proteins are a family of proteins that can become covalently attached to target proteins, much like ubiquitination, in a process called SUMOylation [137,138,139]. There are three different SUMO isoforms, namely SUMO 1, SUMO 2, and SUMO 3, each of which is functionally different from the others [140]. Post-translational modification of target proteins with these SUMO isoforms serves several different downstream functions, including the regulation of protein localization, stability, and protein interactions, as well as cell cycle regulation and proteasomal degradation [137,141,142].

DPCs were first shown to be modified with SUMO when treatment of various mammalian cell lines with camptothecin, a pharmacological inhibitor of topoisomerase 1 resulted in the formation of TOP1-SUMO1 conjugates, while pharmacological treatment with the topoisomerase inhibitor VM-26 resulted in SUMO1 conjugated to both TOP2 isoforms (TOP2α and TOP2β) [92,135,136]. Top2α as well as TOP1 DPCs recovered from etoposide or camptothecin-treated cells, respectively, were shown to be modified with SUMO2/3 [14,91,143]. Similarly, MGMT-DPCs formed following cellular treatment with *N*-methyl-2,2-di(chloroethyl)amine and DNMT1 DPCs formed in various cancer cell lines treated with aza-dC were shown to be SUMOylated [82,84,85]. Studies of post-translational modifications of DPCs formed by formaldehyde treatment showed that while formaldehyde, cisplatin, MMC, HU, IR, and UV treatment all formed DPCs, formaldehyde treatment resulted in the highest amount of total DPCs formed, as well as the largest amount of chromatin SUMOylation [102]. Multiple studies have shown that DPCs formed in formaldehyde-treated cells are modified with SUMO-1 and SUMO-2/3 [95,102]. In one study, nuclear SUMO2/3 foci were not affected by pharmacological inhibition of DNA replication or transcription, suggesting that the system can be used to study the role of SUMOylation in replication and transcription-independent DPC repair [102]. In UV-treated yeast, it was shown that competent SUMO binding was required for TDP1-dependent removal of TOP-1 covalent complexes [100]. In vitro, it was shown that TDP2 removed SUMOylated TOP2βcc more efficiently than total TOP2β. Additionally, it was shown that turnover of the SUMO2-conjugated TOP2β fraction was delayed in Tdp2−/− cells, but only when the proteasome was inhibited, and that TDP2 binds SUMO2, but not SUMO1, suggesting that covalent labeling of TOP2cc with SUMO2 is involved in the recruitment of TDP2 to poisoned TOP2cc [91]. In yeast, it was shown that Wss1 is recruited to SUMOylated targets, and is directly involved in the degradation of Top1cc complexes, in a SUMO-dependent manner [100]. Proteasomal removal of DNMT1-DPCs formed following aza-dC treatment of cancer cells was also SUMO dependent [84]. Similarly, in formaldehyde-treated *C. elegans*, ACRC protease recruitment to formaldehyde-induced foci, was dependent on its SUMO interacting motifs (SIM), showing that there are multiple, SUMO-dependent, and independent mechanisms involved in the repair of formaldehyde-induced DPCs [102].

#### 2.4.3. Poly(ADP-ribose) (PAR)ylation

Poly(ADP-ribose) is a polymer of ADP-ribose moieties synthesized from NAD+ by Poly(ADP-ribose) polymerases (PARPs), and PARylation refers to the process of adding Poly(ADP-ribose) chains to proteins [144,145,146,147]. PARylation serves several important functions in cells, including the recruitment of proteins to trigger protein-protein interactions, apoptosis regulation, and cell signaling [148].

TOP1-DPCS forming in camptothecin-treated cells were rapidly and transiently PARylated (however, the PARylated DPCs were only easily detected when the cells were co-treated with PARGi, a pharmacological inhibitor of dePARylation) [83]. In this study, PARylation was shown to trigger a direct reversal of the topoisomerase–DNA crosslink. Following camptothecin treatment, TDP1 was shown to interact with TOP1 in a PARylation-dependent manner, suggesting that TOP1 PARylation is required for the recruitment of TDP1 to TOP1. Interestingly, this study also showed that PARylation of TOP1-DPCs was hierarchically stronger than TOP1-DPC ubiquitination, as TOP1-DPC PARylation triggered their deubiquitination by USP7, thus blocking recruitment of the proteasome to TOP1-DPCs [83]. While ubiquitination is known to trigger proteasomal degradation of TOP DPCs, PARylation of TOP1-DPCs was also shown to trigger TOP1-DPC de-ubiquitination and in that sense prevent the proteasomal degradation of TOP1-DPCs [83]. The known roles of the above types of post-translational modifications in DPC repair are summarized in Table 1.

### 2.5. Cellular Tolerance or Repair of Enzymatically Processed DPCs 

One common theme of the multiple types of enzymatic processing discussed in Section 2.1, Section 2.2 and Section 2.3 is that the end product still contains lesioned DNA. In some cases, the resulting protein-free DNA contains a double-stranded or single-stranded break in the phosphodiester backbone. In other cases, the initial crosslinked protein has been either proteolytically degraded to yield a peptide fragment crosslinked to the DNA or has been made even larger (by virtue of covalent modification of ubiquitin, SUMO, or PAR). The available evidence indicates that these processed DPCs are, in turn, acted on by a variety of cellular DNA damage tolerance or DNA repair pathways.

While oversized DPCs cause steric hindrance to DNA helicases and replicative polymerases, DPCs that have been subject to proteolytic degradation by SPRTN and/or the proteasome are often tolerated by the cell and do not trigger any further repair mechanisms [94,104,105,149]. Several error-prone DNA polymerases have been identified that are capable of bypassing bulky DNA lesions, including DNA–peptide crosslinks [150,151,152], in a mechanism known as trans-lesion synthesis [152,153,154,155,156,157,158,159]. While DNA-crosslinked proteins that have been ubiquitinated or SUMOylated can ultimately be subject to error-prone trans-lesion synthesis, there is evidence to show that these types of post-translational modifications also target DNA repair proteins that are involved in orchestrating this tolerance response. For example, it has been shown that Rad18-dependent monoubiquitination of proliferating cell nuclear antigen (PCNA) recruits error-prone DNA polymerases to bulky DNA lesions, while PCNA polyubiquitination triggers error-free DNA damage tolerance [160,161,162].

Enzymatically processed DPCs can also be subject to DNA repair. Single-stranded or double-stranded DNA breaks in the DNA backbone following direct reversal of the chemical crosslink can be re-ligated or subject to non-homologous end joining (NHEJ) [59,163]. Small protein DPCs or DNA–peptide crosslinks that result from enzymatic processing of larger proteins crosslinked to DNA are subject to nucleotide excision repair (NER), homologous recombination (HR), or other double-strand break repair pathways [77,80,88,97,117,164,165,166,167]. NER of DPCs is initiated by the recruitment of the transcription factor II H complex to the DNA lesion, followed by incisions at the 5′ end and 3′ end of the DPC lesion, and helicase action, resulting in the release of the DNA-crosslinked oligonucleotide and its surrounding sequence. The resulting single-stranded gap is filled in via DNA polymerase and the nick is sealed by DNA ligase [91,92,93]. HR repair is initiated by the recognition of a double-strand break at the site of nucleolytically processed DPC lesion, followed by 5′ -> 3′ exonuclease action to produce long single-stranded 3′ overhangs, which are then involved in strand invasion of a homologous donor template. Subsequent DNA polymerase-mediated extension of the invading 3′ ends results in Holliday junction formation and resolution [113]. Unlike translesion synthesis, both NER and HR promote high-fidelity DPC resolution. DPC repair by NER and HR has been shown by several groups to be orchestrated by multiple types of enzymatic processing discussed in this review, including proteolytic processing, ubiquitination, and SUMOylation [93,95]. There is also evidence to suggest that other DSB repair pathways, such as NHEJ can repair DSBs formed following nucleolytic processing of DPCs [167,168].

**Table 1:** Role of post-translational modifications in DPC repair.

PTM	Type of DPC	Linkage Determined	Connection to Repair	References
Ubiquitin	TOP1/2	K11, K48, K63	Promotes transcription-dependent, proteasome-dependent repair Recruits TDP2	[14,90,92,169]
	DNMT1	K48	Recruits proteasome during replication-dependent repair	[85]
	HpaII		Triggers proteasomal recruitment in the absence of replication Promotes SUMO-independent repair in the absence of replication	[85,94]
	Formaldehyde induced		Triggers SPRTN-dependent, proteasome-independent repair during S phase progression	[95,102]
	MGMT		Recruits proteasome	[82]
	OGG1	K48, K63	Triggers replication-independent, transcription-independent repair K48 promotes proteasome-dependent repair by NER K63 promotes proteasome-independent repair by HR	[93]
	HMCES		Triggers TLS across DPCs on single-stranded DNA	[170]
	EOS		Triggers unfolding by p97 to facilitate proteolysis by SPRTN	[112]
SUMO	TOP1/2	K7, K11	Triggers ubiquitination and proteasomal degradation SUMO2/3 triggers TDP2 recruitment	[14,91,135,136,169]
	DNMT1		Triggers ubiquitination to recruit proteasome during replication-dependent repair Promotes HR SUMO2/3 triggers ubiquitination via RNF4, and triggers RNF4-independent repair	[84,85,102]
	HpaII		Triggers SPRTN recruitment during replication-independent repair	[94]
	Formaldehyde induced		Recruits ACRC protease SUMO1 promotes SPRTN-dependent, proteasome-independent repair, as well as SPRTN-independent repair SUMO2/3 promotes SPRTN-dependent, proteasome-independent repair	[95,102]
PAR	TOP1		Triggers deubiquitination to block proteasomal processing Triggers TDP1 recruitment	[83]

## 3. Conclusions

As discussed in this review, DPCs are diverse in their size, structure, and chemical identity, and this is reflected in the variety of cellular machinery that can be mobilized to remove them. The DNA component of DPCs can be targeted by nucleases, the chemical crosslink can be targeted by tyrosyl-DNA phosphodiesterase, and the protein component can be targeted by the proteasome, proteases, and multiple covalent modifications (including ubiquitin, SUMO, and PAR). While some types of DPC processing result in full repair of the DPC, most processing results in partial or incomplete repair. It appears likely that multiple types of enzymatic processing of DPCs occur in concert in order to facilitate rapid and efficient DPC repair, and, while the post-translational modifications of DPCs discussed above help us gain an understanding of the signaling that may occur during DPC repair, the exact mechanisms of orchestration of the cellular response to DPCs remains to be understood.

## 4. Therapeutic Implications

As outlined above, several DNA-damaging drugs used in cancer chemotherapy are known to induce DPCs as part of their mechanism of action. Some examples of these drugs include nitrogen mustards, cisplatin, topoisomerase inhibitors like etoposide and camptothecin, and nucleoside analogs like aza-dC. However, cancer cells can develop resistance to these drugs through enhanced ability to repair DPCS. Understanding the mechanisms of DPC repair is vital for addressing resistance and improving the effectiveness of cancer therapies, and identifying cellular machinery involved in DPC processing could be the first step in the identification of new therapeutic targets or strategies. Various studies of the enzymes discussed above that are responsible for catalyzing DPC processing also show that pharmacological or genetic inhibition of these enzymes sensitized cancer cells to DPC-forming drugs (see Table 2). The most successful clinical example is that of PARP1 inhibitors, which have exhibited great efficacy as a monotherapy in the treatment of cancers with defects in DNA repair (specifically the HR pathway), or in combination with radiation or chemotherapy [89,171,172]. However, many pre-clinical studies also show that inhibition of other DPC processing enzymes can sensitize cells to DPC-forming drugs. For example, it has been shown that proteasome inhibition potentiates cancer cell response to DPC-forming drugs [173,174,175,176,177,178]. SPRTN deficiency has also been shown to sensitize cells to DPC-forming drugs [101,179,180]. Inhibitors of ubiquitination and SUMOylation synergized with topoisomerase 1 and topoisomerase 2 poisons [14,90]. Kroonen et al. treated B cell lymphoma cell lines with aza-dC alone, TAK981 (SUMO inhibitor) alone, or both, and showed that the SUMO inhibitor synergized with the aza-dC in eight of the ten cell lines tested. Similarly, in an orthotopic xenograft model, treatment with TAK981 in combination with aza-dC reduced tumor cell growth and increased survival in comparison to either monotherapy, and, as seen in the Sun et al. study, the combination therapy was well tolerated and did not exhibit any increased toxicity [84]. This review also extensively describes how TOP-2 PTM contributes to chemotherapy resistance [181]. Studies have shown that Rad18-induced ubiquitination of PCNA as well as error-prone polymerases play multiple roles in tumorigenesis [182,183]. As biological processes that contribute to oncogenesis are often also involved in the cellular response to anti-cancer drugs, it is unsurprising that a number of studies [184,185] have also shown that altered expression of genes associated with low-fidelity DNA polymerases can also impact cellular sensitivity to cancer chemotherapeutic agents known to produce DPCs. The role of error-prone DNA polymerases is further explored in a number of recent review articles [186,187,188]. Notably, Wang et al. showed that overexpression of the catalytic subunit of the DNA polymerases zeta conferred resistance to cisplatin-but not to other agents that do not induce DPCs in a glioma model [189]. Conversely, suppression of polymerase zeta activity conferred chemotherapeutic sensitivity in a murine lung adenocarcinoma model [190]. Overexpression of functional DNA polymerase eta in human fibroblasts was shown to confer resistance to cisplatin whereas expression of a non-functional variant did not [191]. Notably, Zhou et al. observed a positive correlation between overexpression of DNA polymerase eta and cisplatin resistance in human head and neck squamous cell carcinoma [192]. Together, the studies summarized in this review suggest that pharmacological inhibition of DPC processing could sensitize cancer cells to treatment with DPC-forming drugs, and thus the mechanisms driving these processes have the potential to be targeted to improve clinical outcomes in cancer chemotherapy.

## Figures and Tables

**Table 2:** Enzymes identified in DPC processing.

Enzyme Group	Enzyme	Known Role in DPC Repair	Sensitizes Cells to DPC-Forming Anti-Cancer Drugs	References
Direct Crosslink Removal and Nucleolytic Repair	TDP1	yes	yes	[83,193]
	TDP2	yes	yes	[63,91,169]
	Mre11	yes	unknown	[78,194,195]
	CtIP	yes	yes	[80,194,196]
Proteolytic Repair	Wss1		yes	[100,197]
	SPRTN	yes	yes	[94,95,101,102,103,105,112,180]
	Proteasome	yes	yes	[83,85,87,91,93,173,174,175,176,177,178]
	ACRC	yes	yes	[102]
Covalent Modifications	Ubiquitin-activating Enzyme E1	yes		[14,82,95]
	Ubiquitin-conjugating Enzyme E2			
	E3 ubiquitin-ligase BMi1/Ring1A	yes	yes	[90]
	SUMO Activating Enzyme (SAE)	yes	yes	[14,84,85,95,102,198]
	SUMO ligase ZATT (ZNF451)	yes	yes	[91]
	UBC9 SUMO E2 enzyme		yes	[135]
	Cullin Ring-ubiquitin ligases	yes	yes	[198]
	RFWD3	yes	yes	[170,199]
	RNF4	yes	yes	[14,85]
	PIAS4	yes	yes	[14,85]
	PARP	yes	yes	[83,89,171,172,200]
	Poly(ADP-ribose) glycohydrolase inhibitor	yes	unknown	[83]

## Data Availability

No new data were created or analyzed in this study. Data sharing is not applicable to this article.
